# Comparison of healthspan-related indicators between adults with and without HIV infection aged 18–59 in the United States: a secondary analysis of NAHNES 1999–March 2020

**DOI:** 10.1186/s12889-023-15538-6

**Published:** 2023-05-04

**Authors:** Chen Chen, Xingqi Cao, Jie Xu, Zhen Jiang, Zuyun Liu, Jennifer McGoogan, Zunyou Wu

**Affiliations:** 1 National Center for AIDS/STD Control and Prevention, Chinese Center for Disease Control and Prevention, 155 Changbai Road, Changping District, Beijing, 102206 China; 2 National Institute of Environmental and Health, Chinese Center for Disease Control and Prevention, Beijing, China; 3 Department of Big Data in Health Science, School of Public Health, Zhejiang University School of Medicine, Hangzhou, Zhejiang China; 4 Chinese Center for Disease Control and Prevention, Beijing, China

**Keywords:** HIV, Healthspan, Physical frailty, ADL, Mobility disability, Prevalence

## Abstract

**Background:**

As persons with HIV (PWH) live longer they may experience a heightened burden of poor health. However, few studies have characterized the multi-dimentional health of PWH. Thus, we aimed to identify the extent and pattern of health disparities, both within HIV infection status and across age (or sex) specific groups.

**Methods:**

We used cross-sectional data from the US National Health and Nutrition Examination Survey, 1999–March 2020. The adjusted prevalence of six healthspan-related indicators—physical frailty, activities of daily living (ADL) disability, mobility disability, depression, multimorbidity, and all-cause death—was evaluated. Logistic regression and Cox proportional hazards analyses were used to investigate associations between HIV status and healthspan-related indicators, with adjustment for individual-level demographic characteristics and risk behaviors.

**Results:**

The analytic sample consisted of 33 200 adults (170 (0.51%) were PWH) aged 18–59 years in the United States. The mean (interquartile range) age was 35.1 (25.0–44.0) years, and 49.4% were male. PWH had higher adjusted prevalences for all of the 6 healthspan-related indicators, as compared to those without HIV, ranged from 17.4% (95% CI: 17.4%, 17.5%) vs. 2.7% (95%CI: 2.7%, 2.7%) for all-cause mortality, to 84.3% (95% CI: 84.0%, 84.5%) vs. 69.8% (95%CI: 69.7%, 69.8%) for mobility disability. While the prevalence difference was largest in ADL disability (23.4% (95% CI: 23.2%, 23.7%); *P* < 0.001), and least in multimorbidity (6.9% (95% CI: 6.8%, 7.0%); *P* < 0.001). Generally, the differences in prevalence by HIV status were greater in 50–59 years group than those in 18–29 group. Males with HIV suffered higher prevalence of depression and multimorbidity, while females with HIV were more vulnerable to functional limitation and disabilities. HIV infection was associated with higher odds for 3 of the 6 healthspan-related indicators after fully adjusted, such as physical frailty and depression. Sensitivity analyses did not change the health differences between adults with and without HIV infection.

**Conclusions:**

In a large sample of U.S. community-dwelling adults, by identifying the extent and pattern of health disparities, we characterized the multi-dimentional health of PWHs, providing important public health implications for public policy that aims to improve health of persons with HIV and further reduce these disparities.

**Supplementary Information:**

The online version contains supplementary material available at 10.1186/s12889-023-15538-6.

## Background

With the successful incorporation of combination antiretroviral therapy (ART) into standard clinical practice in the United States (US), persons with HIV (PWH) have experienced increasing life expectancy with more living into old age. Specifically, overall life expectancy for PWH has improved from 59 years in 2000–2003, to 71 in 2008–2010, and 76 in 2014–2016, just 3 years short of the general population (79 in 2014–2016) [1, 2]. Another important gain from ART therapy is that the prognosis of HIV infection has shifted from a terminal disease with a generally poor prognosis to a chronic disease [3, 4]. However, PWH suffered a higher prevalence of non-communicable chronic comorbidities compared with those without HIV individuals [5, 6]. So with high quality of healthspan, which is used to define the length of lifetime with reasonably good health, rather than inflexibly prolonged lifespan has greater practical implications for the vulnerable population (i.e., PWH). Functional impairment, disability, mental health, as well as multi-system conditions were healthspan-related characteristics [7–9]. Increasing attention has been placed on achieving a longer healthspan in the context of dramatic population aging in PWH, where people are spending more years at the older ages and suffering more health problems [8].

So far, a few studies have reported that HIV infection is strongly associated with physical function limitations (ie, physical frailty) [10, 11], worse mental health (ie, depression) [12] and multimorbidity [13, 14]. Physical frailty is an aging-related status characterized by increased vulnerability to minor stressor events causing by cumulative diminished reserve and dysregulation in multiple physiological systems [15]. The prevalence of physical frailty in individuals with HIV ranges from 5 to 28.6% varying by population studied [11]. Age, past opportunistic illnesses, low CD4 counts, longer duration of HIV infection, poorly controlled HIV infection, and advanced course of HIV disease were congruently proved as predictors for frailty [16, 17]. Depression, one of the most prevalent psychiatric disorders, is two- to three-fold more prevalent in PWH than in the general population [18] and is associated with an array of adverse health outcomes, such as poor quality of life, additional comorbidities, disability, and poorer therapeutic outcomes [19]. Among adults with older age and longer ART duration, higher CD4 counts may increase the risk of multimorbidity [20], which has drawn more attentions to these non-HIV related comorbidities among the well-treated PWH [21].

Recently, a study from COmorBidity in Relation to AIDS (COBRA) cohort showed that PWH seem to have an accelerated aging pace with about 9 years “age advancement” (biological minus chronological age) compared with those without HIV counterparters [22]. Moreover, more and more PWH have developed non-AIDS chronic diseases, which have traditionally been associated with aging [23, 24], after receiving cART. Both of above signs suggest that previously mentioned healthspan-related indicators might be contributed by interactions of complex causes, such as antiretroviral treatment, chronic viral co-infections, lifestyle and behavioral risk factors, and accelerated aging process [25, 26].

However, to date, little is known about the multi-dimentional health status among PWHs, such as disability (including activities of daily living (ADL) and mobility disability), especially in nationwide samples of HIV-infected and uninfected populations from developed countries like the US. Furthermore, little is known about whether HIV infection increases health span-related burdens across specific age and sex groups. Therefore, we aimed to identify the extent and pattern of disparities of the six healthspan-related indicators across HIV status overall and stratified by age and sex and to determine whether HIV infection is associated with poorer healthspan-related indicators.

## Methods

### Design

**Fig. 1:**
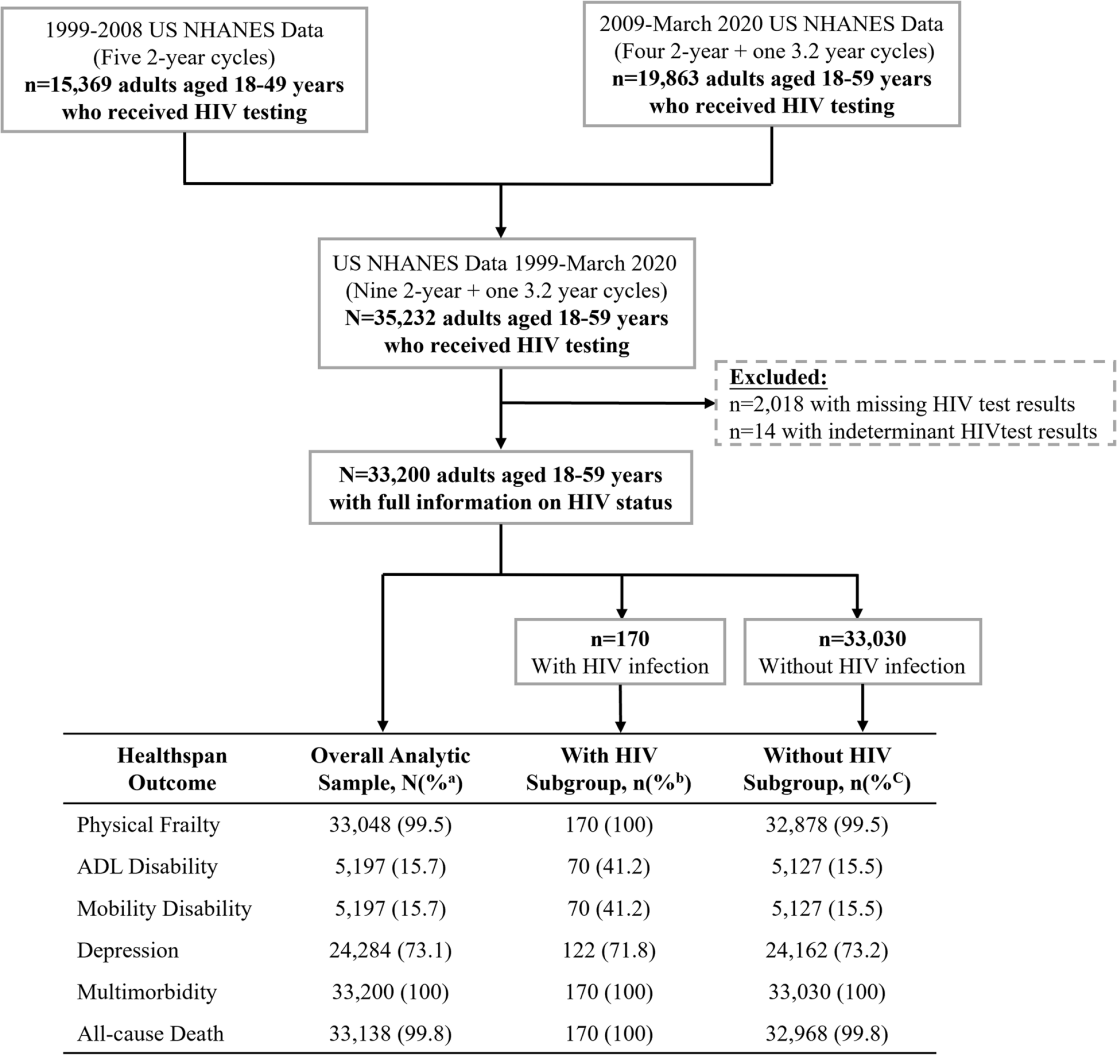
Study design and participants based on adults aged 18–59 from NHANES 1999–March 2020. Abbreviations: ADL, activities of daily living. ^a^ Proportion calculated using *N* = 33,200 (all study participants) as the denominator. ^b^ Proportion calculated using *n* = 170 (all study participants with HIV infection) as the denominator. ^c^ Proportion calculated using *n* = 33,030 (all study participants without HIV infection) as the denominator

This study was designed as a secondary analysis of US National Health and Nutrition Examination Survey (NHANES) data collected from 1999 to March 2020 for adults aged 18–59 years [27], wherein participants were grouped by HIV status and compared across six healthspan outcomes as illustrated in Fig. 1. A total of 33,200 participants were included in the analyses–170 with HIV infection and 33,030 without HIV infection. As this was a secondary analysis of anonymized data in the public domain, no informed consent was required (For detailed data and participants information see Additional File).

### HIV infection status

Participants were grouped by HIV infection status. For HIV testing as a compoenent of the US NHANES comprehensive health screening, serum specimens were first tested using a synthetic peptide enzyme immunoassay assay (EIA) to detect anti-HIV-1 and anti-HIV-2 antibodies. All positive samples were then retested by Western blot (WB) to confirm positive EIA results (Details of analytic notes showed in Additional File).

### Outcomes

The following six healthspan-related indicators were measured in our study:

#### Physical frailty

The functional limitation domain outcome, physical frailty, was measured by a modified version of the Fried physical frailty approach [15]. This measure was developed for NHANES and validated for identification of people at higher risk of functional vulnerability [28, 29]. Participants were categorized as having physical frailty if they met ≥ 3 of the 4 following criteria, i.e., shrinking, weakness, exhaustion and inactivity. Slowness was not included because gait speed was no longer evaluated beginning in the 2003–2004 NHANES cycle.

#### Activities of Daily Living (ADL) disability

The first of two disability domain outcomes, ADL disability included 4 items—dressing, eating, walking, and getting in/out of bed. Participants were categorized as having ADL disability if they had difficulty or needed personal assistance in performing ≥ 1 of these activities.

#### Mobility disability

The second of two disability domain outcomes, mobility disability included seven items—walking for a quarter mile, climbing ten flights of stairs, stooping or kneeling or crouching, getting up from an armless chair, lifting or carrying, reaching up over head, and grasping or holding small objects. Participants were categorized as having mobility disability if they had difficulty or needed personal assistance in performing ≥ 1 of these activities.

#### Depression

The mental health domain outcome, depression, was measured by the Patient Health Questionnaire (PHQ), a version of a self-administered mental disorders diagnostic instrument used in NHANES [30]. The frequency of nine depressive symptoms over the past 2 weeks were self-reported in the PHQ [31]. Each item is scored from 0 (not at all) to 3 (nearly every day) and summed to a total score with a range of 0–27. Individuals with scores of ≥ 5 were categorized as having depression.

#### Multimorbidity

The multisystem condition domain outcome, multimorbidity, was measured as a self-reported count of ten chronic conditions. Based on these disease counts, a variable with five categories was created: 0 through ≥ 4 comorbid conditions (with the last two categories combined in logistic analyses, i.e., with ≥ 3 chronic conditions was defined as having multimorbidity). Notably, HIV disease and AIDS are not included in this multimorbidity outcome count.

#### All-cause death

Mortality was based on linked data in records taken from the National Death Index through December 31, 2019, provided through the US CDC [32]. Mortality status and follow-up time (in person-months) were available for nearly all participants (*N* = 62 with missing data on follow-up time). Further details for the 6 healthspan-related outcomes showed in Additional File.

### Covariates

Five race/ethnicity groups, the poverty income ratio (PIR, below vs. at or above), smoking status (never, former and current), binge drinking (no, yes), and HIV risk behaviors included number of lifetime sexual partners (0–4, 5–9, ≥ 10), lifetime history of same-sex sexual behavior (among men only; no, yes), and lifetime history of injection drug use (no, yes) were used. The detailed criteria for the construction of these covariates were described in Additional File.

### Statistical analyses

Data files from 1999 to March 2020 were combined to conduct these secondary analyses. To obtain population-based estimates, we incorporated a cluster variable, a primary sampling units (PSUs) variable, and a 21.2-year survey weight in the whole analyses (Details showed in Additional File).

Characteristics of participants were summarized using as means and interquartile ranges (IQRs) or frequencies and percentages, and their 95% confidence intervals (CI). Design-based standard error (SE), reflecting variance in the weights, for percentage or prevalence were computed using Taylor series linearization and 95% CIs were computed using the Wald linear confidence limits method [33].

To estimate the adjusted prevalence of healthspan-related indicators by HIV infection status, a marginal structural models method was used, in which an inverse-probability-of-exposure weight that standardizes the population to adjust for confounders based on the original 21.2-year survey weight was constructed [34]. To obtain greater fitness of the inverse probability, we adjusted variables with fewer missing values (ie, age, sex, ethnicity, and education) in the marginal models. An interaction term between HIV infection status (positive, negative) and age (18–29, 30–39, 40–49, 50–59) or sex (male, female) was included in the marginal models to evaluate the adjusted prevalence of healthspan outcomes among specific groups.

Logistic regression models were used to investigate associations between HIV status and healthspan-related indicators, generating odds ratios (ORs) and 95% CIs. Cox proportional hazards models were used to investigate associations between HIV status and all-cause mortality, generating hazard ratios (HRs) and 95% CIs. Four models were considered, each adjusted for different subsets of variables:

- Model 1 – age and sex only;
- Model 2 – model 1, race/ethnicity, education and PIR;
- Model 3 – model 2, smoking, binge drinking, and BMI;
- Model 4 – model 3, lifetime number of sexual partners, and lifetime injection drug use.

Several sensitivity analyses were also performed. First, we estimated the unadjusted prevalence of healthspan-related indicators in age- and sex-specific groups stratified by HIV status. Second, we included slowness in the construction of physical frailty from 1999 to 2002. Third, we further explored the relationship between HIV status and each of the ten chronic diseases, as well as numbers of chronic diseases.

We considered statistical significance if *P*-values were < 0.05 (two-tailed). All analyses conducted using SAS software (v9.4, SAS Institute, Cary, NC, USA).

## Results

### Characteristics of participants

Overall, mean age of 33,200 participants was 35.1 years (IQR: 25.0 – 44.0). As shown in Table 1, most participants overall were female, had more than a high school level education, had a higher PIR, were never-smokers, had recent binge drinking and no lifetime same-sex sexual behavior and lifetime injection drug use. Compared to adults without HIV infection, those with HIV were older (23.1% vs. 15.1% in 50–59 years), had a higher proportion of male (81.3% vs. 49.3%) and non-Hispanic Black (34.2% vs. 10.0%), had lower PIR (34.6% vs. 24.1%), had unhealthier lifestyle (such as current smoker 46.6% vs. 25.0%), more risky sexual behaviors (such as same-sex sexual behaviour in men 85.5% vs 12.2%), and higher comorbidities (23.7% vs. 11.8%).

**Table 1:** Characteristics of participants by HIV infection status among adults aged 18–59 from NHANES 1999–2020

Characteristic	Total (*N* = 33,200)		With HIV (*N* = 170)		Without HIV (*N* = 33,030)	
	**N**	**% (95% CI)**	**N**	**% (95% CI)**	**N**	**% (95% CI)**
Age category, year						
18–29	12 318	30.9 (29.9—32.0)	30	13.2 (7.3—19.0)	12 288	31.0 (29.9—32.1)
30–39	8 385	26.3 (25.6—27.1)	46	22.1 (13.9—30.3)	8 339	26.4 (25.6—27.1)
40–49	8 304	27.6 (26.8—28.3)	57	41.7 (29.2—54.1)	8 247	27.5 (26.7—28.3)
50–59	4 193	15.2 (14.3—16.0)	37	23.1 (14.0—32.1)	4 156	15.1 (14.3—15.9)
Sex						
Male	15 765	49.4 (48.8—50.0)	126	81.3 (75.7—87.0)	15 639	49.3 (48.7—49.9)
Female	17 435	50.6 (50.0—51.2)	44	18.7 (13.0—24.3)	17 391	50.7 (50.1—51.3)
Race/Ethnicity						
Non-Hispanic White	11 472	50.1 (47.5—52.6)	27	35.4 (21.8—49.0)	11 445	50.1 (47.6—52.7)
Non-Hispanic Black	5 531	10.1 (8.9—11.2)	82	34.2 (25.0—43.5)	5 449	10.0 (8.8—11.1)
Mexican American	8 128	23.7 (21.8—25.6)	14	10.0 (3.3—16.6)	8 114	23.8 (21.9—25.6)
Hispanic	3 639	8.1 (7.3—9.0)	5	3.2 (0.0—6.6)	3 634	8.2 (7.3—9.0)
Other Race	4 430	8.0 (7.1—8.9)	42	17.2 (11.4—23.0)	4 388	7.9 (7.0—8.9)
Education level^a^						
< High school	6 768	14.9 (14.0—15.8)	43	18.3 (11.5—25.1)	6 725	14.9 (14.0—15.8)
High school or equivalent	8 016	24.2 (23.1—25.2)	38	22.2 (13.1—31.4)	7 978	24.2 (23.2—25.2)
> High school	16 828	61.0 (59.4—62.5)	86	59.4 (47.8—71.1)	16 742	61.0 (59.4—62.5)
Poverty index ratio^a^						
Below PIR	10 538	24.1 (22.8—25.4)	72	34.6 (24.6—44.6)	10 466	24.1 (22.8—25.3)
At or above PIR	19 952	75.9 (74.6—77.2)	82	65.4 (55.4—75.4)	19 870	75.9 (74.7—77.2)
Smoking status^a^						
Never	15 842	56.7 (55.5—57.9)	60	38.5 (24.8—52.2)	15 782	56.8 (55.6—58.0)
Former	4 343	18.3 (17.4—19.1)	21	14.9 (7.2—22.6)	4 322	18.3 (17.4—19.1)
Current	6 799	25.1 (24.0—26.1)	71	46.6 (34.8—58.5)	6 728	25.0 (23.9—26.0)
Binge drinking^a^						
No	3 420	8.5 (7.6—9.4)	16	7.8 (3.2—12.5)	3 404	8.5 (7.6—9.4)
Yes	27 095	91.5 (90.6—92.4)	151	92.2 (87.5—96.8)	26 944	91.5 (90.6—92.4)
BMI, kg/m^2 ab^	32 812	27.4 (23.6—32.1)	166	25.5 (22.7—28.5)	32 646	27.4 (23.6—32.1)
Number of sexual partners lifetime^a^						
0–4	10 102	39.8 (38.6—41.0)	19	10.1 (5.0—15.2)	10 083	39.9 (38.7—41.1)
5–9	5 792	25.1 (24.4—25.9)	26	17.0 (10.8—23.3)	5 766	25.2 (24.4—26.0)
> = 10	7 882	35.1 (34.0—36.2)	85	72.9 (64.7—81.0)	7 797	34.9 (33.8—36.0)
Ever had same-sex sexual behavior (men only)						
No	4 498	86.9 (85.2—88.5)	16	14.5 (6.9—22.2)	4 482	87.8 (86.2—89.5)
Yes	592	13.1 (11.5—14.8)	62	85.5 (77.8—93.1)	530	12.2 (10.5—13.8)
Ever used illicit or injection drugs^a^						
No	22 398	78.7 (77.9—79.6)	90	56.2 (45.9—66.5)	22 308	78.8 (78.0—79.7)
Yes	5 207	21.3 (20.4—22.1)	62	43.8 (33.5—54.1)	5 145	21.2 (20.3—22.0)
Multicomorbidity						
Without	29 538	88.1 (87.5—88.8)	127	76.3 (66.8—85.8)	29 411	88.2 (87.5—88.9)
With	3 662	11.9 (11.2—12.5)	43	23.7 (14.2—33.2)	3 619	11.8 (11.1—12.5)

Note: No. was based on study samples (unweighted). Column percentages and their 95% CI were weighted population estimates, which were incorporated a cluster variable, a primary sampling units (PSUs) variable, and a 16-year survey weight variable in the analyses

*Abbreviations*: *CI* confidence intervals, *IQR* interquartile range, *BMI *body mass index

^a^Numbers of missing data ranged from 388 to 9424 (1588 for education level, 2710 for poverty index ratio, 6216 for smoking status, 2685 for binge drinking, 388 for BMI, 9424 for the number of sexual partners lifetime, and 5595 for ever used illicit or injection drugs)

^b^Values are given as median and IQR, calculated from weighted population estimates

### Healthspan-Related indicators by HIV Infection Status

The analytic sample sizes were varied by healthspan-related indicators, ranging from 5,197 (15.7%, for ADL and mobility disability) to 33,200 (100%, for multimorbidity) accordingly (Fig. 1).

For all of the 6 healthspan-related indicators, PWH had higher adjusted prevalences as compared to those without HIV (Table 2). The adjusted prevalences ranged from 17.4% (95% CI: 17.4%, 17.5%) in PWH vs. 2.7% (95% CI: 2.7%, 2.7%) in those without HIV for all-cause mortality, to 84.3% (95% CI: 84.0%, 84.5%) in PWH vs. 69.8% (95% CI: 69.7%, 69.8%) in those without HIV for mobility disability. However, the prevalence difference between persons with vs. without HIV was largest in ADL disability (23.4% (95% CI: 23.2%, 23.7%); *P* < 0.001), and least in multimorbidity (6.9% (95% CI: 6.8%, 7.0%); *P* < 0.001).

**Table 2:** Adjusted prevalences and population estimates of health outcomes in the United States 1999-March 2020, by HIV infection status

**Outcomes**		**Persons with HIV**			**Persons without HIV**		**Prevalence Difference,** **% (95%CI)**	*P*-Value^*^
	**n/N**^**a**^	**Prevalence**^**b**^**,** **% (95%CI)**	**Estimated Population**^**c**^	**n/N**^**a**^	**Prevalence**^**b**^**,** **% (95%CI)**	**Estimated Population**^**c**^		
Physical Frailty	50/170	28.9 (28.8, 29.0)	330 147	3 705/32 878	10.4 (10.4, 10.4)	28 419 884	18.5 (18.4, 18.6)	< 0.001
ADL Disability	36/70	59.7 (59.5, 60.0)	246 540	2 014/5 127	36.3 (36.3, 36.3)	16 514 816	23.4 (23.2, 23.7)	< 0.001
Mobility Disability	59/70	84.3 (84.0, 84.5)	392 823	3 689/5 127	69.8 (69.7, 69.8)	31 757 450	14.5 (14.2, 14.8)	< 0.001
Depression	52/122	41.9 (41.7, 42.0)	414 507	5 725/24 167	22.4 (22.4, 22.4)	47 676 267	19.5 (19.3, 19.6)	< 0.001
Multimorbidity	43/170	18.9 (18.8, 19.0)	280 525	3 619/22 030	12.0 (12.0, 12.0)	32 465 944	6.9 (6.8, 7.0)	< 0.001
All-cause Death	29/170	17.4 (17.4, 17.5)	179 588	961/32 968	2.7 (2.7, 2.7)	7 443 135	14.7 (14.6, 14.8)	< 0.001

*Abbreviations*: *CI* 95% confidence interval, *ADL* activities of daily living

^a^Number for both numerator and denominator were based on unweighted study samples

^b^Prevalence, % and (95% CI) were calculated by marginal structural models using an inverse-probability-of-exposure weight adjusted for age, sex, ethnicity and education

^c^Refers to weighted number of n case, which were calculated from weighted estimates, eg., 50 represented 330,147 population with HIV suffered with physical frailty, 3,705 represented 28,419,884 population without HIV suffered with physical frailty; Weighted numbers of N population were also calculated, eg., 170 represented 1,184,357 population with HIV, 32,878 represented 273,794,954 US adults

^*^P value was obtained from the marginal structural model method (see in the Analyses section) between with HIV vs. without HIV

### Healthspan-related indicators by age- and sex-specific HIV status

**Fig. 2:**
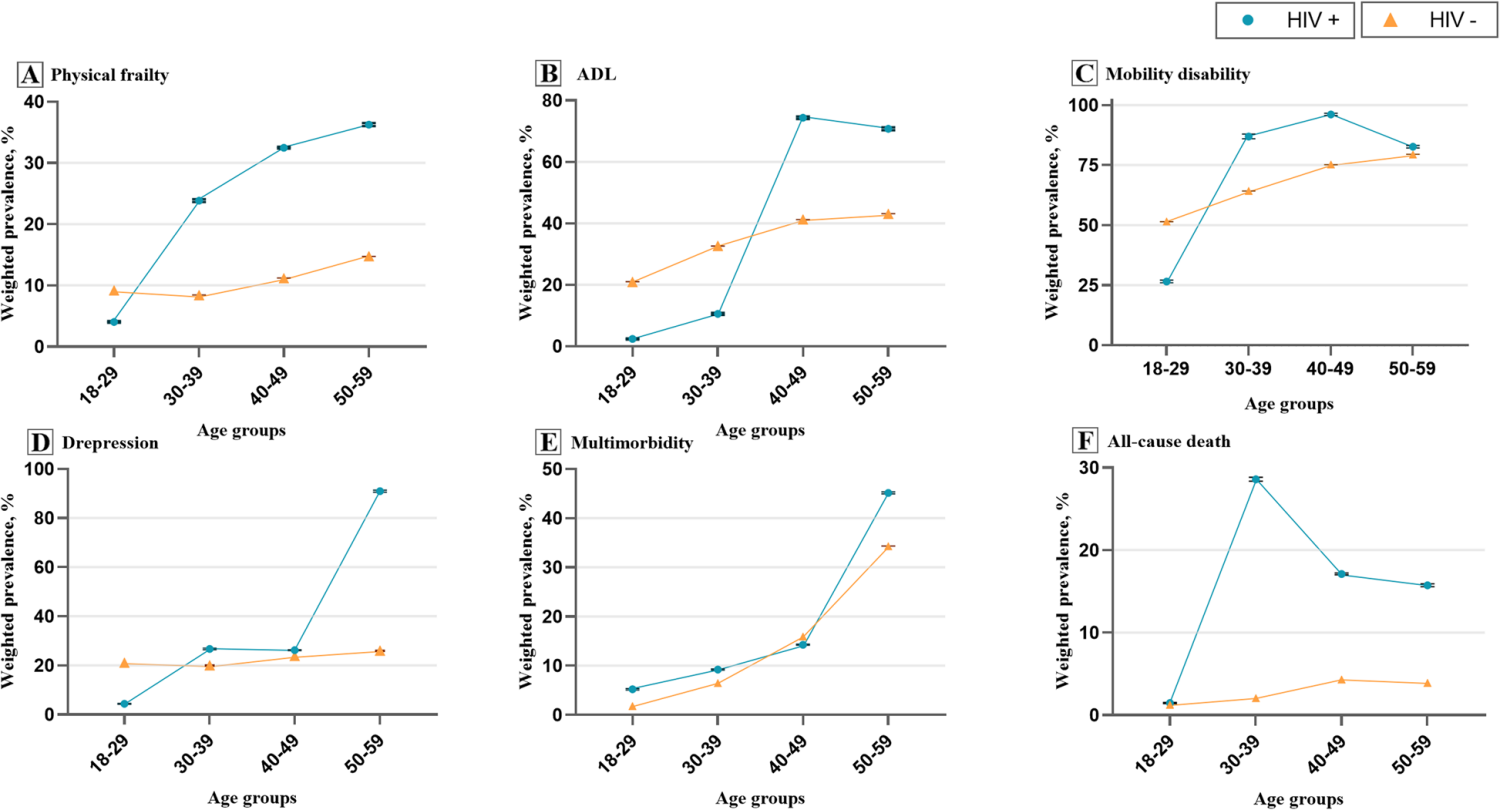
Adjusted Prevalence^a^ of Healthspan-related Indicators Regarding Age-specific HIV Status among Adults from NHANES 1999-March 2020. NHANES, the National Health and Nutrition Examination Survey; ADL, activities of daily living. ^a^ Adjusted for age, sex, ethnicity and education. Error bars showed the 95% confidence intervals (CIs). Noting that the 95% CIs of HIV negative group was too narrow so the error bars could not been seen

Figure 2 showed the adjusted prevalence of the 6 outcomes for each age group in HIV infection status. Overall, the adjusted prevalence in adults without HIV group (orange line) presented a flat level among the four age groups for the 6 healthspan-related indicators; while in PWH group (blue line), the adjusted prevalences were similarly at higher levels than the without HIV group among the four age groups for all outcomes; except for *multimorbidity*, as the prevalences were comparable between HIV among all age groups. Noted that NHANES data did not provide temporal trends.

We found that in 18–29 years group, prevalences were lower in HIV-positive group for functional limitation, disability, and depression, such as 4.05% (95% CI: 3.92%, 4.17%) in PWH vs. 9.20% (95% CI: 9.20%, 9.21%) in those without HIV for *physical frailty* (data showed in Supplementary Table S1). On the other hand, in 50–59 years group, prevalences were higher in PWH for the 6 outcomes except for *mobility disability*, such as 36.26% (95% CI: 36.00%, 36.52%) in adults with HIV vs. 14.77% (95% CI: 14.76%, 14.78%) in adults without HIV for *physical frailty*. Generally, the prevalence gaps between HIV were greater in 50–59 years group than those in 18–29 group for all healthspan-related indicators, except for mobility disability.

**Fig. 3:**
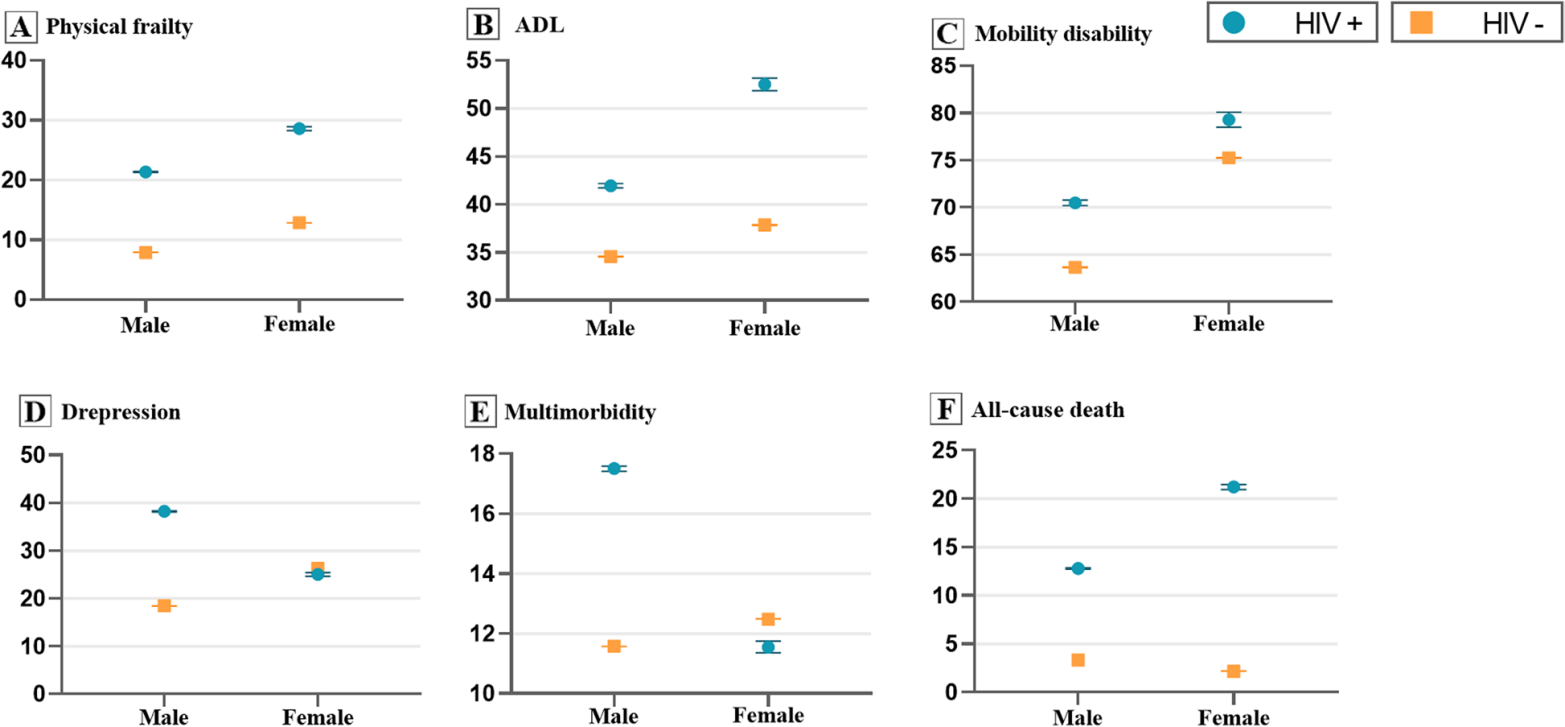
Adjusted Prevalence^a^ of Healthspan-related Indicators Regarding Sex-specific HIV Status among Adults from NHANES 1999-March 2020. NHANES, the National Health and Nutrition Examination Survey; ADL, activities of daily living. ^a^ Adjusted for age, sex, ethnicity and education. Error bars showed the 95% confidence interval (CIs). Noting that the 95% CIs of HIV negative group was too narrow so the error bars could not been seen

Figure 3 showed the adjusted prevalences of the 6 healthspan-related indicators for each sex group in HIV infection status. Generally, we observed that prevalence gaps were greater for most outcomes in male than in female except for *ADL disabiliy and all-cause death*; and males with HIV suffered higher prevalences of *depression* and *multimorbidity* while females with HIV were more vulnerable to *functional limitation and disabilities* (data showed in Supplementary Table S2).

### Associations of HIV infection status with healthspan-related indicators

**Fig. 4:**
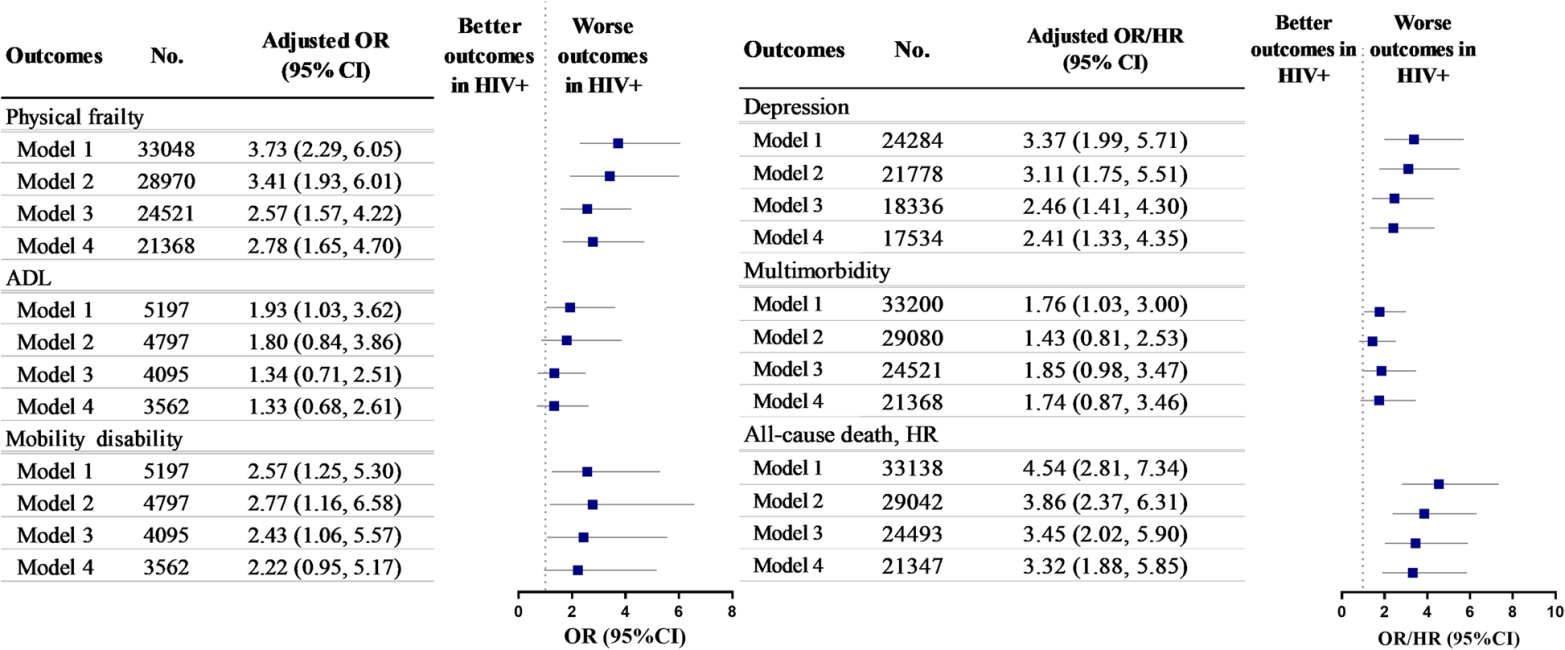
Associations of HIV Infection Status with Healthspan-related Indicators among adults aged 18–59 from NHANES 1999-March 2020. NHANES, the National Health and Nutrition Examination Survey; ADL, activities of daily living. Model 1 adjusted for age and sex; model 2 further adjusted for race/ethnicity, education and PIR; model 3 further adjusted for smoking, binge drinking status and BMI based on model 2; model 4 further adjusted for lifetime number of sexual partners and lifetime injection drug use based on model 3

As shown in Fig. 4, after fully adjusted for age, sex, ethnicity, education, PIR, smoking, binge drinking status, BMI, lifetime number of sexual partners and lifetime injection drug use, HIV infection was associated with nearly threefold higher odds for prevalent physical frailty (OR, 2.78; 95% CI: 1.65, 4.70), depression (OR, 2.41; 95% CI: 1.33, 4.35), and all-cause mortality (HR, 3.32 95% CI: 1.88, 5.85), respectively; no associations were observed with functional disability and multimorbidity.

### Sensitivity analyses

In sensitivity analyses, we found that: (1) the general patterns of the unadjusted prevalence for all six outcomes in age- and sex-specific HIV groups were unchanged; health burden gaps of adults with vs. without HIV were still with the highest among older age (50–59 years, Supplementary Fig. S1) except for all-cause mortality, and males with HIV had higher rates of depression while females with HIV were more suffered with physical limitations (Supplementary Fig. S2); (2) included gait speed included to construct an alternative type of physical frailty with 5 items from 1999 to 2002 did not substantially change the associations of HIV with physical frailty appreciably (OR, 2.36; 95% CI: 1.07, 5.22 in adjustment model 4; Supplementary Table S3); (3) PWHs were more likely to report prevalent liver disease, kidney disease, hepatitis C infection, cancer, kidney disease, and hypertension; 19.0% in adults with HIV had co-exist two or three comorbidities compared to 10.6% in those without HIV (Supplementary Table S4).

## Discussion

Using data of 33,200 adults aged 18 to 59 years who participated in HIV antibody testing for the U.S. NHANES survey, we characterized the multi-dimentional health status of persons with HIV infection. We found that the prevalence difference between persons with and without HIV group was largest in ADL disability, and least in multimorbidity; health gaps of adults with vs. without HIV were larger in older ages; males with HIV had higher prevalent depression and multimorbidity, while females with HIV were more suffered with physical limitation and disabilities. Overall, multimorbidity did not differ by HIV infection.

In this nationally representative sample of household U.S. adults, 28.9% and 41.9% HIV-infected adults met the criteria for physical frailty and depression, respectively. The prevalence estimate for physical frailty is in line with a recent literature review [11], which reported that the ranges prevalent physical frailty from 5 to 28.6% in community-based HIV infected adults aged 20–70 years worldwide (10 studies from the U.S., 1 from Mexico and 1 from South Africa). We could not ignore the influence of the absence of gait speed, one of the important components for physical frailty to assess individual’s functional mobility, while which is might partly be represented by mobility disability. Nevertheless, this prevalence is remarkable when it comes to the large population of HIV-infected adults in the U.S. (i.e., an estimate of 1.2 million HIV-infected people aged 13 and older in the United States at the end of 2018). That being said, approximately 35 thousand and 50 thousand HIV-infected adults may additionally suffer physical frailty and depression, respectively, in the U.S., and may require urgent and considerable attention.

Disability among the HIV-infected population is increasingly recognized but data are limited. Disability is usually assessed by self-report of difficulty in completing specific tasks, rather than the objective measurements of physical function. ADL refer to the basic and essential skills needed to properly take care of oneself, for example dressing, eating, walking, and getting in/out of bed [35]. While mobility disability affects movement ranging from gross motor skills, such as walking, to fine motor movement, involving manipulation of objects by hand, which assess higher level of physical function [36]. Disability in ADL and mobility may lead to dependence on others or mechanical devices, unsafe conditions, and poor quality of life [37]. In the early AIDS epidemic of the pre-ART era (1988–1991), among 728 HIV-infected patients, 18% reported prevalent ADL disability only, 14% reported prevalent instrumental activities of daily living (IADL) diability only and 4% in both ADL and IADL disability [38]. In the era of effective ART, only four studies investigated the associations between HIV infection and prevalent disability status, which found that the prevalences of disability in ADL and mobility among HIV-infected adults ranged from 19 to 92%, and 28% to 81%, respectively [39]. The prevalence estimates for HIV-infected household U.S. adults in this study are 59.7% for ADL disability and 84.3% for mobility disability, respectively, which is within in the range above. Disability could seriously deteriorate the quality of life, which would further increases the difficulties of HIV patient management.

Studies showed that improvements on the continued reductions in mortality and morbidity among HIV-infected adults in North America and Europe, which might obscure the notable negative effects when survival data and health data are stratified by age, where older infected individuals suffered inferior responses to ART therapy and beared higher burdens of chronic disease [40]. All of these may lead to the study of healthspan-related indicators in HIV-infected populations, especially in older HIV-infected ones. In our study, we found that the health gaps between persons with vs. without HIV were higher in older ages. Some reasons may explain the age discrepancies: 1) Older adults could be more likely to be diagnosed with HIV later than youngers, where 49% of adults aged 60 years and older progressed to AIDS in 1 year since they were diagnosed with HIV infection during 2009 compared with 14% of adults younger than 25 years [41]. 2) Besides the late diagnosis of HIV infection, responses to ART therapy (e.g., CD4 count) in older patients were consistently inferior to they were presented in young adults (consistent with our results as presented in Table S1), which might be associated with the mediated immune reconstitution that depends on the thymic function. However, the reconstruction function decreases with aging [42]. 3) Combination drug use of antiretroviral agents and other drugs are common in older adults, so older HIV-infected individuals may be exposed at higher risk of adverse events [43]. Furthermore, the toxicity of polypharmacy interactions could not be ignored [44].

Sex specific patterns of healthspan-related indicators showed that males with HIV had higher prevalent depression and multimorbidity, while females with HIV more suffered from physical limitation and disabilities. Studies found that bone density and bone loss are affected by the interaction of HIV infection and estrogen levels. Further, bone mineral density assessments for patients with fragility fractures are advocated for all HIV-infected postmenopausal women [45]. Noting that an underlying etiology pathway might be associated with the sex specific characteristic of nutritional and hormonal factors and the remarkably prevalent physical limitation and disabilities in females [46]. Public health resource is needed to be appropriately allocated for physical function screening for HIV infected female and depression screening among HIV infected male in secondary prevention.

In the current study, data from a nationally representative sample of household adults in the U.S. and the availability of data on multiple healthspan-related indicators provided us a unique opportunity to disoict the multi-dimentional health of PWHs, of which some (e.g., ADL and mobility disability) have not been well investigated previously. Second, we presented the prevalence by adjusted for multiple demographic variables, which could balance the disparities between HIV infected and uninfected populations. Third, we explored the extent and patterns of health disparities, both within HIV infection status and stratified by age or sex, which provided more detailed health burden estimates at targeted subgroups for public policymakers aimed at reducing these disparities.

The current study nevertheless has several limitations. First, due to the NHANES sampling design aimed to gain a nationally representative sample of household U.S. adults, the numbers of adults with HIV infection and some age groups (such as 18–29, and 50–59 years) are relatively small, which may lead to false-positive results. However, we provided the weighted number of PWH in U.S., which will be useful to estimate overall healthcare sources needed in future studies. Second, several important confounders, such as CD4 counts, CD8 counts were not included in our analyses (only 156 adults have CD4/CD8 values). We admitted missing in CD4 or CD8 is an obvious shortage for understanding the mechanism of comorbidities among persons with HIV, but they are not confounding factors when we report the adjusted prevalence. Third, the ten diseases for construction of multimorbidity varied in studies, which might influence the prevalence estimates and associations analyses to some extent. However, we report the selected chronic illnesses that have strong biological and clinical etiological associations with HIV infection which may strengthen our findings [40]. Fourth, the unexpected highly mortality in 30–39 years old adults with HIV among both crude and adjusted models need to be reconsidered in future national-wide research with larger population of persons with HIV.

## Conclusion

In a large sample of U.S. community-dwelling adults, we found that the prevalence gaps between persons with vs. without HIV are substantially and significantly larger in the older adults for most healthspan-related indicators. Males with HIV had higher prevalence of depression and multimorbidity, while HIV-infected females were more likely to suffer from physical limitation and disabilities. Overall, multimorbidity did not differ by HIV infection. These age- and sex-specific findings have important public health implications for public policy making which is aiming at improving the health of persons with HIV and further reducing these disparities.

## Supplementary Information

**Additional file 1.** Detailed information about the Methods and Results section. And Supplementary Figure S1—S2 and Supplementary Table S1—S4.

## Data Availability

Deidentified data were available in the NHANES website (https://wwwn.cdc.gov/nchs/nhanes/continuousnhanes/default.aspx). The qualitative data used and/or analysed during the current study are available from the corresponding author on reasonable request.

## Abbreviations

NHANES: National Health and Nutrition Examination Survey
PWH: Persons with HIV
ADL: Activities of daily living
ART: Antiretroviral therapy
US: United States
COBRA: COmorBidity in Relation to AIDS
EIA: Enzyme immunoassay assay
WB: Western blot
PSUs: Primary sampling units
IQRs: Interquartile ranges
CI: Confidence intervals
BMI: Body mass index
