# Supplementary material for: Comparison of healthspan-related indicators between adults with and without HIV infection aged 18–59 in the United States: a secondary analysis of NAHNES 1999–March 2020
Source: BMC Public Health. 2023 May 4;23:814. doi: 10.1186/s12889-023-15538-6 (PMC10157932; doi:10.1186/s12889-023-15538-6)
Supplement: Supplementary file 1 — Additional file 1. Detailed information about the Methods and Results section. And Supplementary Figure S1—S2 and Supplementary Table S1—S4. [file 12889_2023_15538_MOESM1_ESM.docx]

**Additional File**

**Methods**

**Data**

NHANES is an ongoing cross-sectional survey that collects data from ~5,000 participants every year using a complex, multistage probability cluster sampling design to produce a nationally representative sample of the community-dwelling US population. Datasets are publicly released in 2-year cycles. Each 2-year cycle contains data from ~10,000 individuals collected via structured interview and comprehensive health screening. Among them, ~3,000 individuals per cycle provided venous blood samples for HIV testing. Beginning in 2009, in recognition of the aging being observed among people with HIV-positive status, NHANES expanded the age range of adults included in HIV testing from 18–49 to 18–59 years [1].

**Participants**

All NHANES participant records in the nine 2-year and one 3.2-year cycles from 1999 to March 2020 were screened for study eligibility. Inclusion criteria were [a] being an adult aged 18–59 at time of participation and [b] having been tested for HIV as part of the NHANES health screening. Individuals who had HIV test results that were missing or indeterminant were excluded. Among the 35,232 NHANES participants from 1999 to March 2020 who received HIV testing, 2,018 were excluded for missing results and 14 for indeterminate results. Thus, a total of 33,200 participants were included in the analyses--170 with HIV infection and 33,030 without HIV infection (**Figure 1**).

**HIV infection status**

The HIV antibody result was coded as negative if the Synthetic Peptide Enzyme Immunoassay Assay (EIA) was repeatedly negative, or if the EIA was positive or indeterminate but the Western blot (WB) was negative; coded as positive if both EIA and WB were positive; and coded as indeterminate if the EIA was positive or indeterminate but the WB was indeterminate. CD4 and CD8 counts were available only among 1999-2006 cycles.

**Outcomes**

*Physical Frailty:* Participants were categorized as having physical frailty if they met ≥ 3 of the 4 following criteria. First, shrinking was established as a self-reported ≥10 pound or ≥ 5% unintentional weight loss in the previous year or having a BMI ≤ 18.5 kg/m^2^. Second, weakness was defined as a self-report of difficulty when lifting or carrying 10 pounds. Third, exhaustion was determined by a self-report of difficulty walking from one room to another on the same level without using special equipment. Fourth, inactivity was established as a self-report of being less active compared to same-age and same-sex peers.

*Depression:* The frequency of nine depressive symptoms over the past 2 weeks were self-reported in the PHQ, which is comprised of five cognitive/affective depressive symptoms (ie, lack of interest, depressed mood, worthlessness, concentration problems, and suicidal ideation) and four somatic depressive symptoms (i.e., sleep difficulties, fatigue, appetite problems, psychomotor agitation, and retardation) [2].

*Multimorbidity:* The multisystem condition domain outcome, multimorbidity, was measured as a self-reported count of ten chronic conditions including: liver disease, kidney disease, hepatitis C virus infection, congestive heart failure, stroke, cancer, arthritis, type 2 diabetes, hypertension, and myocardial infarction.

**Covariates**

Five race/ethnicity groups (non-Hispanic white, non-Hispanic black, other race (including non-Hispanic multi-racial), Mexican American, and Hispanic) were constructed. The poverty income ratio (PIR) is an index for the ratio of family income to poverty. The PIR was calculated by dividing family income by the poverty guidelines according to the participant's household size, as well as the specific year and state. Below PIR and above PIR with the cutoff point at 1.3 were categorized [3]. Smoking status was categoried into three levels, i.e., never (<100 cigarettes during one’s lifetime), former (100 or more cigarettes during one’s lifetime, but not actively smoking recently), and current (ongoing smoking habit). A binary binge drinking indicator was constructed, which was defined as having 5 and more alcoholic beverages at a time at least once per month; otherwise, defined as no binge drinking. HIV risk behaviors included number of lifetime sexual partners (0–4, 5–9, ≥10), lifetime history of same-sex sexual behavior (among men only; no, yes), and lifetime history of injection drug use (no, yes).

**Statistical Analyses**

Following NHANES analytic guidelines [4-5], a 21.2-year survey weight was created as follows: first, multiply the 4-year sample weight for 1999-2002 by 2, then divide the doubled 4-year 1999-2002 sample weight and the 2-year weights for the 2003-2010 cycles, by 6, the number of cycles; second, the 2017–March 2020 prepandemic data file covers 3.2 years compared with 2 years for other NHANES data files, so a 3.2-year survey weights should be adjusted when 2017–March 2020 data files are combined with other 2-year cycles. Multiply the 2-year sample weight for 2011-2016 by 2 and multiply the 3.2-year sample weight for 2017- March 2020 by 3.2, then divide by 9.2 (the number of cycles 2011 to March 2020); the resulting sample weight will be a 21.2-year weight.

**References**

1. Woodring J, Kruszon-Moran D, McQuillan G. HIV Infection in U.S. Household Population Aged 18-59: Data From the National Health and Nutrition Examination Survey, 2007-2012. Natl Health Stat Report. 2015(83):1-13.

2. Leavens A, Patten SB, Hudson M, Baron M, Thombs BD. Influence of somatic symptoms on Patient Health Questionnaire-9 depression scores among patients with systemic sclerosis compared to a healthy general population sample. Arthritis Care Res (Hoboken). 2012;64(8):1195-201.

3. Arshad T, Golabi P, Paik J, Mishra A, Younossi ZM. Prevalence of Nonalcoholic Fatty Liver Disease in the Female Population. Hepatol Commun. 2019;3(1):74-83.

4. National Health and Nutrition Examination Survey: Analytic Guidelines, 1999-2010. [Available from: https://wwwn.cdc.gov/nchs/data/nhanes/analyticguidelines/99-10-analytic-guidelines.pdf]

5. National Health and Nutrition Examination Survey, 2017–March 2020 Prepandemic File: Sample Design, Estimation, and Analytic Guidelines. [Available from: https://www.cdc.gov/nchs/data/series/sr_02/sr02-190.pdf].

**Table S1 Adjusted prevalence ^†^ of different health span-related outcomes by age-specific HIV status among adults in the United States, NHANES 1999-March 2020.**

| **Age groups** | **Outcomes** | **With HIV** | **Without HIV** | **With vs. Without** |
| --- | --- | --- | --- | --- |
|  |  | **Prevalence, % (95% CI)** | **Prevalence, % (95% CI)** | **Prevalence difference, % (95% CI)** |
| 18-29 | Physical frailty | 4.05 (3.92, 4.17) | 9.20 (9.20, 9.21) | 5.16 (5.03, 5.28)**^*^** |
|  | ADL disability | 2.41 (2.25, 2.58) | 20.98 (20.95, 21.01) | 18.57 (18.40, 18.73)**^*^** |
|  | Mobility disability | 26.59 (26.04, 27.14) | 51.53 (51.48, 51.58) | 24.94 (24.39, 25.49)**^*^** |
|  | Depression | 4.38 (4.21, 4.55) | 21.12 (21.11, 21.13) | 16.74 (16.57, 16.91)**^*^** |
|  | Multimorbidity | 5.22 (5.08, 5.36) | 1.80 (1.80, 1.81) | 3.42 (3.28, 3.56) |
|  | All-cause death | 1.46 (1.38, 1.54) | 1.26 (1.26, 1.27) | 0.20 (0.12, 0.27) |
| 30-39 | Physical frailty | 23.85 (23.63, 24.07) | 8.43 (8.43, 8.44) | 15.42 (15.19, 15.64) |
|  | ADL disability | 10.55 (10.23, 10.88) | 32.69 (32.65, 32.72) | 22.14 (21.81, 22.46) **^*^** |
|  | Mobility disability | 86.95 (86.02, 87.89) | 64.28 (64.23, 64.33) | 22.67 (21.74, 23.61) |
|  | Depression | 26.70 (26.34, 27.06) | 20.00 (19.99, 20.01) | 6.70 (6.34, 7.06) |
|  | Multimorbidity | 9.23 (9.09, 9.36) | 6.49 (6.48, 6.49) | 2.74 (2.61, 2.87) |
|  | All-cause death | 28.59 (28.35, 28.83) | 2.06 (2.06, 2.06) | 26.53 (26.29, 26.77) |
| 40-49 | Physical frailty | 32.49 (32.34, 32.65) | 11.19 (11.18, 11.20) | 21.30 (21.15, 21.46) |
|  | ADL disability | 74.38 (73.97, 74.79) | 41.28 (41.25, 41.31) | 33.10 (32.69, 33.51) |
|  | Mobility disability | 96.11 (95.65, 96.57) | 75.23 (75.18, 75.27) | 20.88 (20.42, 21.35) |
|  | Depression | 26.17 (26.01, 26.34) | 23.87 (23.86, 23.89) | 2.30 (2.13, 2.46) |
|  | Multimorbidity | 14.29 (14.20, 14.39) | 15.94 (15.93, 15.95) | 1.65 (1.55, 1.74)**^*^** |
|  | All-cause death | 17.12 (17.01, 17.23) | 4.31 (4.31, 4.32) | 12.81 (12.69, 12.92) |
| 50-59 | Physical frailty | 36.26 (36.00, 36.52) | 14.77 (14.76, 14.78) | 21.49 (21.23, 21.75) |
|  | ADL disability | 70.75 (70.27, 71.23) | 43.12 (43.08, 43.16) | 27.63 (27.15, 28.11) |
|  | Mobility disability | 82.63 (82.12, 83.15) | 79.46 (79.41, 79.51) | 3.17 (2.65, 3.69) |
|  | Depression | 90.97 (90.54, 91.40) | 26.00 (25.98, 26.02) | 64.97 (64.54, 65.40) |
|  | Multimorbidity | 45.15 (44.93, 45.36) | 34.33 (34.32, 34.35) | 10.81 (10.60, 11.03) |
|  | All-cause death | 15.73 (15.55, 15.90) | 3.91 (3.90, 3.92) | 11.82 (11.64, 11.99) |

Note: Prevalence and 95% CIs were calculated from weighted population estimates. All prevalence differences between with vs. without HIV infection groups were all at P<0.001 using the Rao-Scott Chi-Square.

NHANES, the National Health and Nutrition Examination Survey; ADL, basic activities of daily living.

^†^ Adjusted for age, sex, ethnicity and education.

^*^ The adjusted prevalence were higher in without HIV group vs. with HIV group.

**Table S2 Adjusted prevalence ^†^ of different health span-related outcomes by sex-specific HIV status among adults in the United States, NHANES 1999-March 2020.**

| **Sex groups** | **Outcomes** | **With HIV** | **Without HIV** | **With vs. Without** |
| --- | --- | --- | --- | --- |
|  |  | **Prevalence, % (95% CI)** | **Prevalence, % (95% CI)** | **Prevalence difference, % (95% CI)** |
| Male | Physical frailty | 21.35 (21.26, 21.44) | 7.88 (7.87, 7.88) | 13.47 (13.38, 13.56)**^*^** |
|  | ADL disability | 41.95 (41.73, 42.18) | 34.57 (34.55, 34.60) | 7.38 (7.16, 7.61) **^*^** |
|  | Mobility disability | 70.49 (70.20, 70.79) | 63.65 (63.62, 63.68) | 6.85 (6.55, 7.14)**^*^** |
|  | Depression | 38.23 (38.09, 38.38) | 18.46 (18.45, 18.47) | 19.78 (19.63, 19.92)**^*^** |
|  | Multimorbidity | 17.51 (17.42, 17.59) | 11.58 (11.57, 11.58) | 5.93 (5.85, 6.02)**^*^** |
|  | All-cause death | 12.79 (12.71, 12.86) | 3.32 (3.32, 3.33) | 9.46 (9.39, 9.53)**^*^** |
| Female | Physical frailty | 28.59 (28.29, 28.89) | 12.88 (12.88, 12.89) | 15.71 (15.41, 16.01)**^*^** |
|  | ADL disability | 52.52 (51.87, 53.17) | 37.85 (37.83, 37.88) | 14.67 (14.02, 15.32)**^*^** |
|  | Mobility disability | 79.28 (78.48, 80.08) | 75.25 (75.22, 75.29) | 4.02 (3.22, 4.83)**^*^** |
|  | Depression | 25.03 (24.64, 25.42) | 26.33 (26.32, 26.34) | 1.30 (1.69, 0.91)**^*^** |
|  | Multimorbidity | 11.55 (11.36, 11.75) | 12.48 (12.48, 12.49) | 0.93 (1.12, 0.74)**^*^** |
|  | All-cause death | 21.21 (20.95, 21.47) | 2.17 (2.17, 2.17) | 19.05 (18.79, 19.31)**^*^** |

Note: Prevalence and 95% CIs were calculated from weighted population estimates. All prevalence differences between with vs. without HIV infection groups were all at P<0.001 using the Rao-Scott Chi-Square.

NHANES, the National Health and Nutrition Examination Survey; ADL, basic activities of daily living.

^†^ Adjusted for age, sex, ethnicity and education.

^*^ The adjusted prevalence were higher in without HIV group vs. with HIV group.

**Table S3 Associations of HIV Infection Status with Alternatived Physical Frailty among adults aged 18-49 in the United States, NHANES 1999-2002.**

| **Physical Frailty** | **No.** | **OR (95%CI) ^†^** |
| --- | --- | --- |
| Model 1 | 3 816 | 1.97 (1.02, 3.83) |
| Model 2 | 3 557 | 2.25 (1.09, 4.63) |
| Model 3 | 3 179 | 2.39 (1.08, 5.31) |
| Model 4 | 2 980 | 2.36 (1.07, 5.22) |

NHANES, the National Health and Nutrition Examination Survey.

^†^Model 1 adjusted for age and sex; model 2 further adjusted for race/ethnicity, education and PIR; model 3 further adjusted for smoking, binge drinking status and BMI based on model 2; model 4 further adjusted for sexual behavior factors (i.e., number of sexual partners lifetime and ever used illicit or injection drug) based on model 3.

**Table S4 Distribution of the multimorbidities by HIV infection status among adults aged 18-59 in the United States, NHANES 1999-March 2020.**

| **Outcomes** | **Total (N=33 200)** | | **With HIV (N=170)** | | **Without HIV (N=33 030)** | |
| --- | --- | --- | --- | --- | --- | --- |
|  | **N** | **%(95 % CI)** | **N** | **%(95 % CI)** | **N** | **%(95 % CI)** |
| **Self-reported disease history** |  |  |  |  |  |  |
| Congestive heart failure | 273 | 0.8 (0.6 - 0.9) | 2 | 1.5 (0.0 - 3.9) | 271 | 0.7 (0.6 - 0.9) |
| Stroke | 364 | 1.1 (1.0 - 1.2) | 2 | 0.8 (0.0 - 1.8) | 362 | 1.1 (1.0 - 1.2) |
| Cancer or malignancy | 985 | 4.3 (4.0 - 4.7) | 12 | 7.7 (2.1 - 13.3) | 973 | 4.3 (4.0 - 4.7) |
| Arthritis | 3 845 | 14.3 (13.6 - 14.9) | 26 | 14 (6.8 - 21.2) | 3 819 | 14.3 (13.6 - 14.9)* |
| Diabetes | 2 120 | 6.1 (5.7 - 6.5) | 15 | 6.8 (2.5 - 11.2) | 2 105 | 6.1 (5.7 - 6.5) |
| High blood pressure | 6 089 | 19.3 (18.5 - 20.0) | 54 | 32.8 (22.6 - 43.0) | 6 035 | 19.2 (18.5 - 20.0)* |
| Heart attack | 358 | 1.1 (1.0 - 1.3) | 3 | 2.2 (0.0 - 4.9) | 355 | 1.1 (1.0 - 1.3) |
| Liver disease | 902 | 3 (2.7 - 3.2) | 28 | 14 (7.5 - 20.5) | 874 | 2.9 (2.7 - 3.2)* |
| Weak/failing kidneys | 504 | 1.6 (1.4 - 1.8) | 16 | 11.1 (4.0 - 18.2) | 488 | 1.5 (1.3 - 1.7)* |
| Hepatitis C infection | 371 | 2.3 (2.0 - 2.6) | 12 | 10.9 (2.8 - 18.9) | 359 | 2.3 (2.0 - 2.6)* |
| **Multicomorbidity** |  |  |  |  |  |  |
| None | 22 888 | 66.0 (65.1 - 66.9) | 80 | 45.3 (35.1 - 55.5) | 22 808 | 66.1 (65.2 - 67.0)* |
| 1 | 6 650 | 22.1 (21.4 - 22.8) | 47 | 31 (21.7 - 40.4) | 6 603 | 22.1 (21.4 - 22.7) |
| 2 | 2 412 | 8.1 (7.6 - 8.6) | 23 | 13.9 (5.3 - 22.5) | 2 389 | 8.1 (7.5 - 8.6) |
| 3 | 839 | 2.6 (2.3 - 2.8) | 11 | 5.1 (1.6 - 8.6) | 828 | 2.5 (2.3 - 2.8) |
| 4+ | 411 | 1.2 (1.0 - 1.4) | 9 | 4.6 (1.6 - 7.7) | 402 | 1.2 (1.0 - 1.4) |

NHANES, the National Health and Nutrition Examination Survey.

^*^ P<0.01, Rao-Scott Chi-Square.


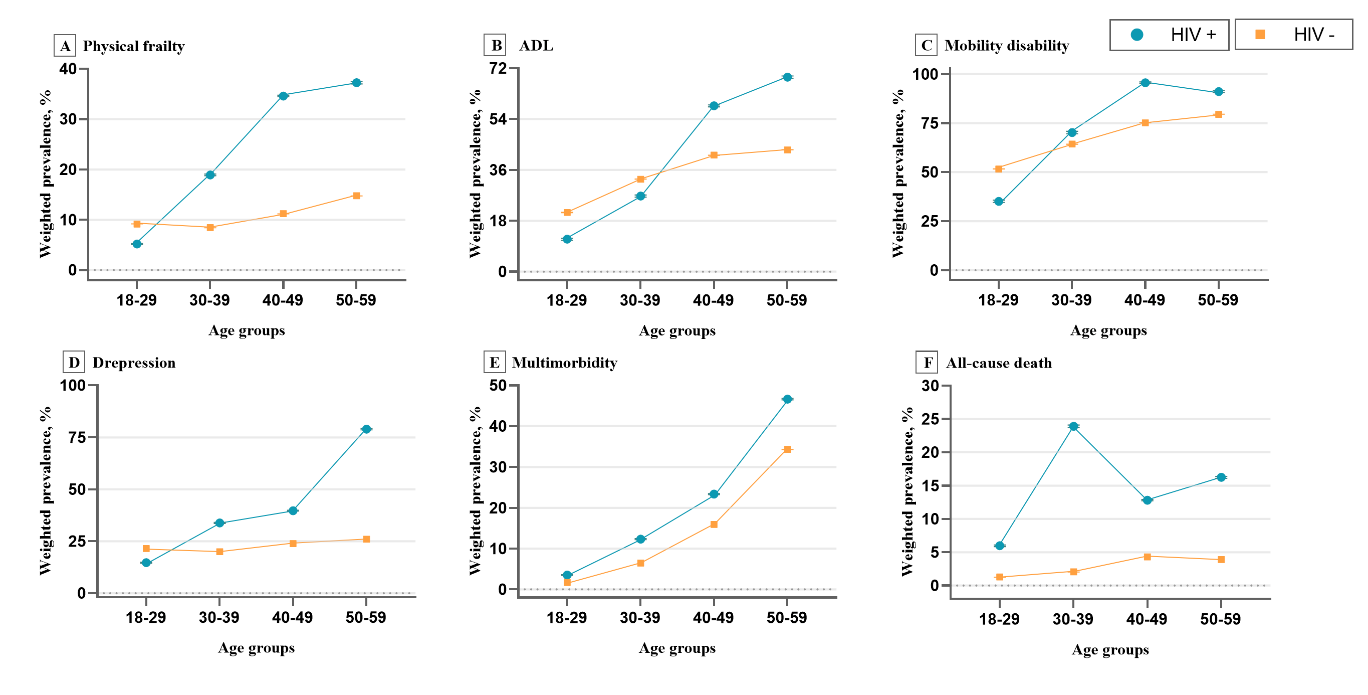


**Figure S1. Cruded Prevalence of Healthspan-related Outcomes Compared by Age-specific HIV Status among Adults in the United States, NHANES 1999-March 2020.** NHANES, the National Health and Nutrition Examination Survey; ADL, basic activities of daily living. Error bars showed the 95% confidence intervals (CIs). Noting that the 95% CIs of HIV-positive group was too wide so the error bars were not shown.


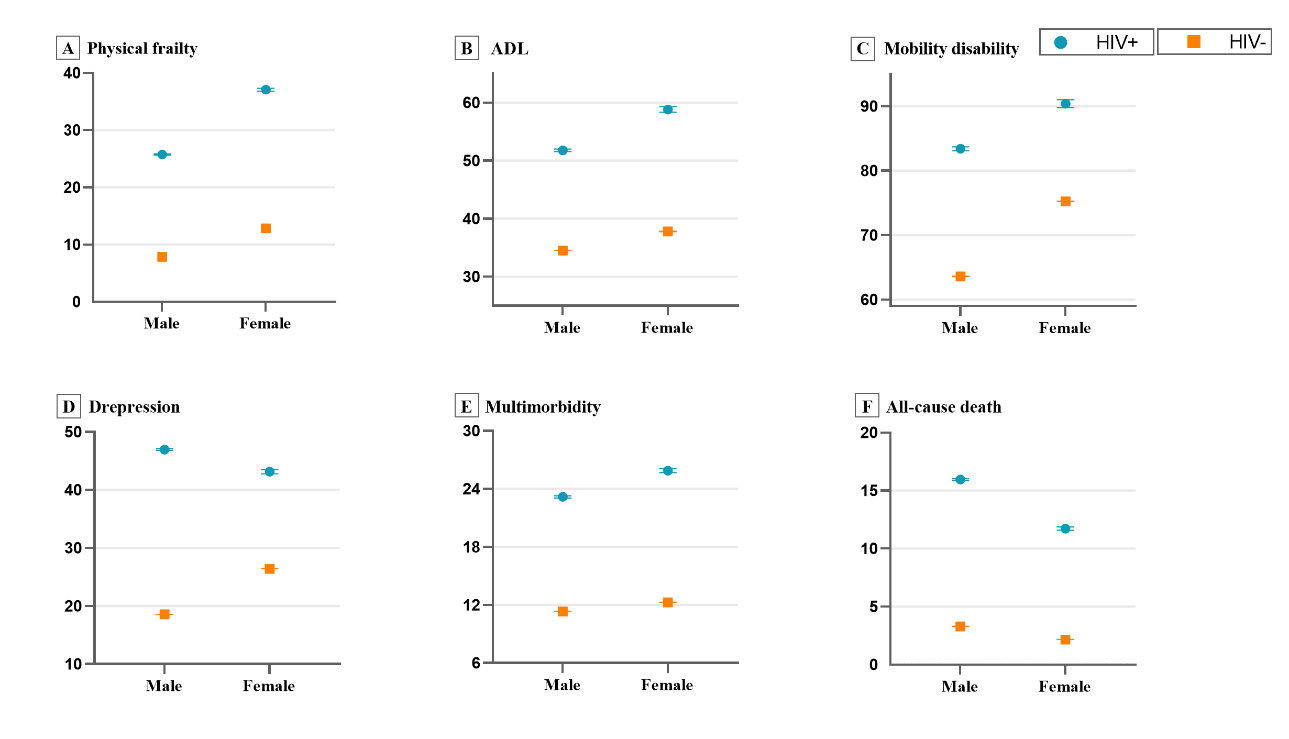


**Figure S2. Cruded Prevalence of Healthspan-related Outcomes Compared by Sex-specific HIV Status among Adults in the United States, NHANES 1999-March 2020.** NHANES, the National Health and Nutrition Examination Survey; ADL, basic activities of daily living. Error bars showed the 95% confidence intervals.
